# Large-scale super-resolution optoacoustic imaging facilitated by FeNP/ICG-loaded coreless polyelectrolyte microcapsules

**DOI:** 10.7150/thno.112050

**Published:** 2025-05-25

**Authors:** Daniil Nozdriukhin, Shuxin Lyu, Daniel Razansky, Xosé Luís Deán-Ben

**Affiliations:** 1 Institute for Biomedical Engineering and Institute of Pharmacology and Toxicology, Faculty of Medicine, University of Zurich, Winterthurerstrasse 190, Zurich, 8057 Switzerland; 2 Institute for Biomedical Engineering, Department of Information Technology and Electrical Engineering, ETH Zurich, Wolfgang-Pauli-Strasse 27, Zurich, 8093 Switzerland; 3 Institute of Medical Technology, Shanxi Medical University, 030001 Taiyuan, China

**Keywords:** Localization Optoacoustic Tomography, Microcapsules, Layer-by-Layer, Brain, Testis, Tumor

## Abstract

**Rationale:** Localization optoacoustic tomography (LOT) enhances imaging of deep-tissue microvasculature by leveraging flowing contrast particles. However, achieving high-resolution, large-scale imaging requires contrast agents with strong per-particle signal, good biocompatibility, and prolonged circulation time. This study introduces coreless polyelectrolyte microcapsules (MCs) encapsulating indocyanine green (ICG) and iron oxide nanoparticles (FeNP) to overcome current limitations in LOT imaging.

**Methods:** MCs were engineered using a layer-by-layer technique by depositing polyelectrolytes, FeNP and ICG on a CaCO_3_ core, which was eventually dissolved. Their optical, morphological, and biocompatibility properties were characterized via UV-Vis-NIR spectroscopy, SEM, and toxicity assays *In vitro* and *In vivo*. Optoacoustic tomography (OAT), motion contrast optoacoustic imaging (MC-OA), directional motion contrast optoacoustic imaging (DMC-OA), localization optoacoustic tomography (LOT), and velocity mapping were conducted in mice to evaluate cerebral, testicular, and tumor vasculature. A raster scanning approach enabled large-scale brain imaging with 9-position coverage.

**Results:** MCs displayed strong optoacoustic contrast, low cytotoxicity, and long circulation times (>45 min). *In vivo* LOT imaging revealed super-resolved microvascular networks in brain, testis, and tumor, with up to 2.5-fold enhancement in vessel visualization parameters. Velocity maps enabled quantification of cerebral blood flow, and oxygenation maps were further rendered by integrating LOT with spectral unmixing. Extended imaging was enabled by persistent MC signal, facilitating full-cortex vascular imaging.

## Introduction

Optoacoustic (photoacoustic) tomography (OAT) is gaining maturity as a powerful angiographic tool, providing unparalleled capabilities for high-resolution, label-free visualization of vascular structures deep within biological tissues 1-5. The method capitalizes on the high optical absorption properties of hemoglobin in red blood cells (RBCs), resulting in excellent vascular contrast. However, the OAT's angiographic imaging performance has been constrained by two key limitations. On the one hand, the small size and high density of RBCs in blood practically result in continuous absorption of blood vessels for typical spatial resolutions achieved with standard systems 6. As a result, arbitrarily oriented vascular networks can only be accurately resolved if the emitted wavefield is fully captured with sufficient angular coverage, an impractical condition in most realistic imaging scenarios 7-9. On the other hand, the spatial resolution of OAT has been fundamentally limited by acoustic diffraction, which hinders visualization of microvasculature in deep tissues 10,11. Blood pool microparticulate contrast agents can overcome these limitations by effectively breaking the continuity of absorption contrast, thus rendering speckled images with enhanced tomographic visibility 12. The contrast fluctuations induced by flowing particles can additionally be exploited for super-resolution imaging 13,14. In particular, localization optoacoustic tomography (LOT) generates super-resolution images by mapping isolated particle positions and quantifying velocity through particle tracking 15. The *In vivo* feasibility of LOT has recently been established, breaking the conventional trade-offs between resolution and depth in OAT 16-18.

Nonetheless, advancing microparticles as contrast agents for LOT and, more broadly, for particle-enhanced OAT remains a challenge. A key requirement is that these particles flow smoothly through the vasculature, including capillary networks, without causing microvessel blockage. As a result, the practical size is limited to a few micrometers with softer materials being preferable to ensure seamless flow. Additionally, microparticles should incorporate a high concentration of light-absorbing (contrast) materials to generate sufficiently strong per-particle OAT signals. Structural and contrast materials of the particles must also be biocompatible for pre-clinical studies, with FDA-approved substances being preferred to facilitate an eventual clinical translation. Moreover, it is important to take into that LOT image resolution, accuracy, and overall quality heavily rely on the number of localized particles. The circulation time of microparticles in blood is thus of high importance, particularly if a large area needs to be covered by acquiring data at different positions. It is also important to consider that the light-absorption-based contrast mechanism of OAT offers great versatility, enabling e.g. visualization of a wide range of dyes or nanoparticles targeting specific tissues, thereby complementing microparticle-based super-resolution angiographic images 19-25.

Various types of microparticles have been suggested as LOT contrast agents, including formulations using FDA-approved indocyanine green (ICG) as light-absorbing material 16-18,26-28. Moreover, many other particle types could potentially exploit the versatility of OAT contrast to achieve enhanced LOT performance. Among these, layer-by-layer (LbL)-based polyelectrolyte microcapsules (MCs) stand out for their design flexibility, allowing fine-tuning of size, surface charge, and composition 29-33. This type of particles is created through controlled deposition of alternating polyelectrolyte layers, forming a shell structure capable of encapsulating functional agents such as nanoparticles or dyes. Their coreless nature enhances biocompatibility by mimicking cellular structures, ensuring improved integration into biological systems, reduced toxicity, and a safer overall profile for *In vivo* applications. In this work, we developed coreless LbL-based polyelectrolyte MCs loaded with iron oxide nanoparticles (FeNP) and ICG as contrast agents for enhancing OAT contrast, visibility, and resolution. The strong per-particle signals and prolonged circulation time of the MCs is shown to enable rendering of high-quality super-resolution LOT images, as well as other types of images offering multiparametric contrast. The good performance of this imaging methodology is demonstrated in vascularized tissues such as the brain, the testis, and subcutaneous tumors. Also, the acquisition of OAT image sequences at different scanning positions within the circulation time of the MCs is shown to allow for detailed visualization of the cortex-wide mouse brain vasculature.

## Results

### Synthesis and characterization of coreless microcapsules

**Figure 1:**
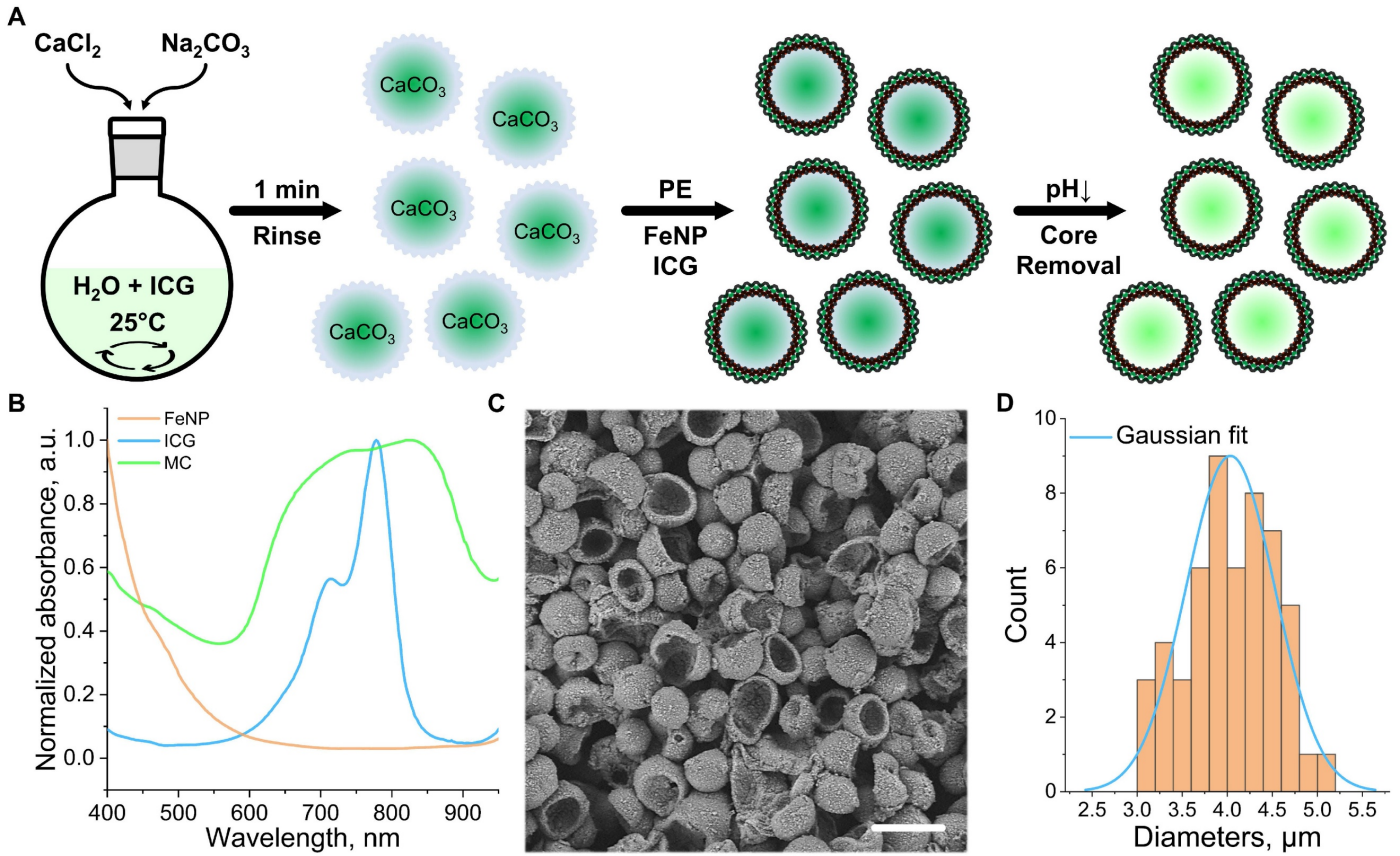
** Preparation and structural analysis of coreless polyelectrolyte microcapsules (MCs).** (A) Schematic representation of the MC synthesis procedure. The process involves the synthesis of indocyanine green (ICG)-loaded calcium carbonate (CaCO₃) cores, coating of these with layers of polyelectrolytes (PE), iron nanoparticles (FeNP), and ICG, and core dissolution in a low-pH medium. (B) UV-Vis-NIR spectra of the MCs (green line) and their components: ICG (blue line) and FeNP (orange line). (C) SEM micrograph of the synthesized MCs. Scalebar - 5 µm. (D) Size distribution of the MCs, determined from SEM data (n = 50 particles, orange histogram) and corresponding Gaussian fit (blue line).

Shell-loaded (coreless) polyelectrolyte MCs were synthesized using a two-step procedure (Figure 1A, see methods for details). First, mesoporous ICG-loaded calcium carbonate (CaCO_3_) cores in the vaterite form and with a size range of 3-5 µm were prepared, rinsed to remove residual reactants and unattached ICG, and coated with two bilayers of polyelectrolyte cushion composed of poly(diallyldimethylammonium chloride) (PDDA) and poly(sodium styrene sulfonate) (PSS), denoted as ([PDDA/PSS]₂). This coating prevents the recrystallization of vaterite in subsequent steps and enhances the loading capacity of the shells. Four layers of iron oxide nanoparticles (FeNPs) were subsequently deposited using PDDA as interlayers, resulting in a structure denoted as CaCO₃/[PDDA/PSS]₂/[PDDA/FeNP]₄. This modification increases shell roughness, improves structural stability, and contributes to OA signal generation via light absorption (Figure S1). Subsequently, four bilayers of ICG and PSS ([ICG/PSS]₄) were added to boost the MCs' near-infrared (NIR) absorption. A protective coating of two bilayers of [PDDA/PSS] was ultimately applied to stabilize the MCs. This final coating reduces ICG photodegradation and renders a negative surface charge to minimize fouling in biological fluids 34,35.

In the second step, the vaterite cores were dissolved using a hydrogen chloride (HCl) solution (pH = 3), resulting in the formation of hollow MCs. UV-Vis-NIR spectroscopy confirmed the presence of ICG within the MC structure (Figure 1B). The observed broadening of the ICG absorption peak is attributed to aggregation of the dye within the shell, influenced by residual NaCl and oxidation under reduced pH conditions during the core dissolution process. FeNPs incorporated into the shell contribute to increasing its porosity, thereby enhancing the ICG loading capacity. This was demonstrated by measuring the absorbance of the supernatant after successive shell adsorption steps, with shell saturation occurring at a later adsorption cycle when FeNPs are present (Figure S2A). Additional photothermal imaging experiments showed that, in the absence of FeNPs, the MC signal shows degradation after the second heating cycle under continuous-wave (CW) laser illumination (1 W/cm², 795 nm, Figure S2B-D). Scanning electron microscopy (SEM) images revealed the MCs' spherical shape and rough-textured morphology, the latter being associated with metallic inclusions (Figure 1C). The small diameter (4.0 ± 0.5 µm, Figure 1D), estimated based on measurements from 50 MCs in SEM images, and the flexibility of the MCs facilitate seamless flow through microvascular pathways, minimizing the risk of capillary arrest.

### Cell toxicity and biocompatibility

**Figure 2:**
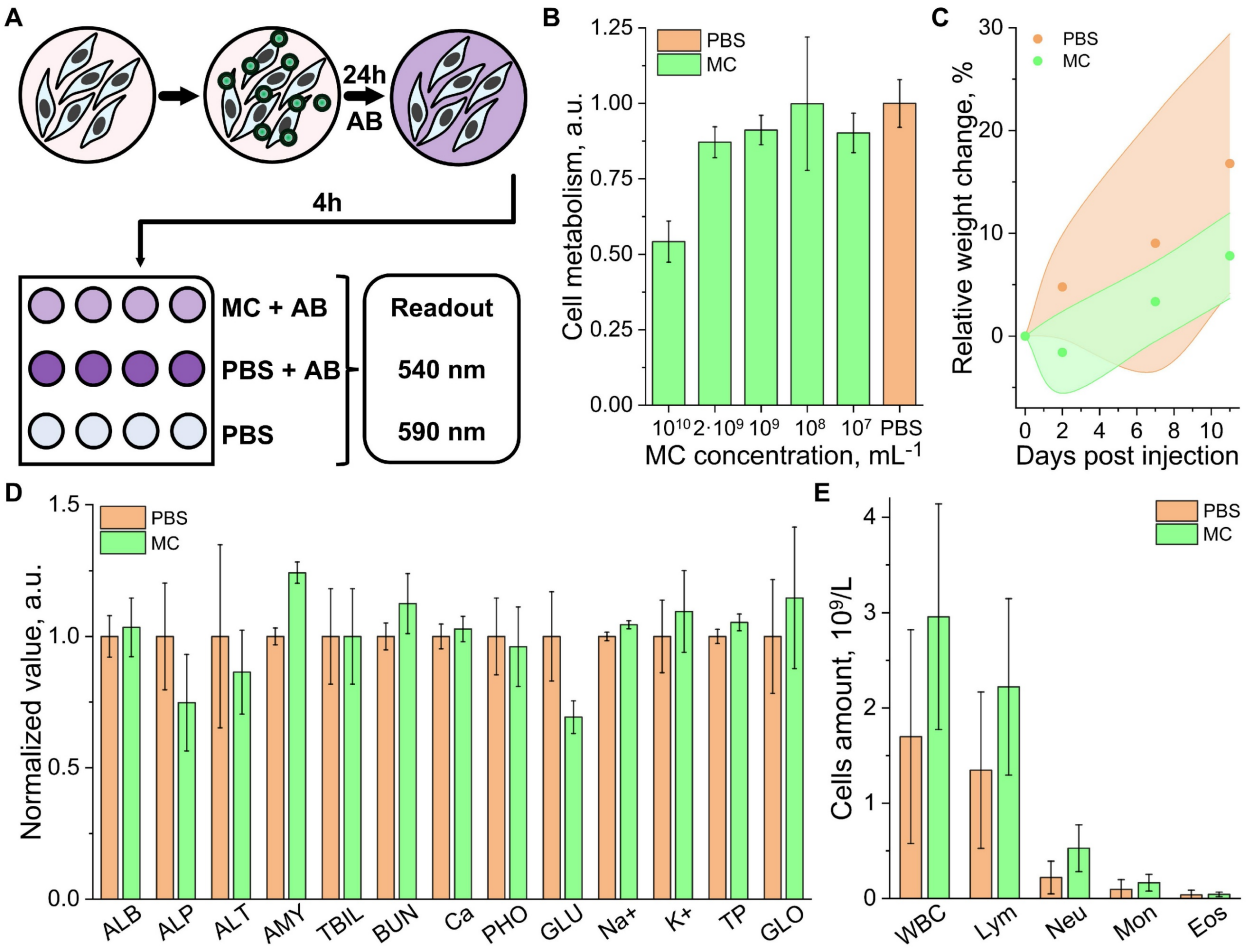
** Cytotoxicity and biocompatibility aassessment.** (A) Schematic illustration of the AlamarBlue (AB) cell metabolism assay: cells are incubated with microcapsules (MCs) for 24 hours, followed by a 4-hour incubation with AB. Fluorescence readouts are taken at an excitation wavelength of 540 nm and an emission wavelength of 580 nm. The signal from cells, supplemented with phosphate-buffered saline (PBS) was used as a control. (B) Results of the AB cell viability assay on Chinese hamster ovary (CHO) cell cultures treated with varying concentrations of MCs. (C) Weight evolution of mice involved in the biocompatibility study (n = 4 per group; 2 males, 2 females), measured over 11 days post-injection of MCs or PBS. The shaded areas represent the range (minimum and maximum) of recorded weights. (D) Murine blood biochemistry on day 11 post-injection of MCs or PBS. Parameters include: ALB (albumin), ALP (alkaline phosphatase), ALT (alanine transaminase), AMY (amylase), TBIL (total bilirubin), BUN (blood urea nitrogen), CA (calcium), PHOS (phosphates), GLU (glutamine), Na⁺ (sodium), K⁺ (potassium), TP (total protein), and GLOB (globulin). (E) Immune cell profile of murine blood on day 11 post-injection of MCs or PBS. Parameters include: WBC (total white blood cells), Neu (neutrophils), Mon (monocytes), and Eos (eosinophils).

The biocompatibility and acute toxicity of the MCs were evaluated both *In vitro* and *In vivo*. The dose resulting in cellular damage was determined by co-incubating the MCs at various concentrations (10⁷ to 10¹⁰ particles/mL) with Chinese hamster ovary (CHO) cell cultures for 24 hours (Figure 2A, see methods for details). Cell viability and metabolism were assessed using an AlamarBlue assay, as the most toxic effects typically manifest within the first 24 hours (also taking into account CHO exponential doubling time is 20-24h) 36,37. The assay demonstrated good cell viability for concentrations below 2 × 10⁹ particles/mL (Figure 2B), i.e., within the range required for individual per-particle detection *In vivo* 26.

A cell-safe concentration of 10⁹ particles/mL was further tested in Swiss mice (8 weeks old). The study included 2 groups of 4 mice (2 males and 2 females per group), which received an intravenous (i.v.) injection of 100 µL boluses of a suspension of MCs and a control phosphate-buffered saline (PBS), respectively. Mice weights were monitored throughout the experimental timeline (Figure 2C), and blood samples were collected on the 11th day for analysis. Biochemical analyses revealed no significant deviations from physiological norms in either group. The slight changes in glutamine (GLU) and amylase (AMY) levels observed in the MC-injected group may be attributed to mild inflammation but remained far below levels associated with toxic effects. Similar levels of alkaline phosphatase (ALP), alanine transaminase (ALT), and blood urea nitrogen (BUN) were measured in both groups, ruling out significant kidney or liver damage. Total bilirubin (TBIL) and total protein (TP), associated with red blood cell (RBC) lysis, also remained within the same range for both groups (Figure 2D). Hematology analysis showed a slight elevation in immune cell counts in the study group, which may indicate a mild immune response (Figure 2E). However, no significant changes in body weight, physical condition, or behavior were observed in any of the mice during the study period, ruling out any significant adverse effect.

### Multiparametric super-resolution optoacoustic tomography

**Figure 3:**
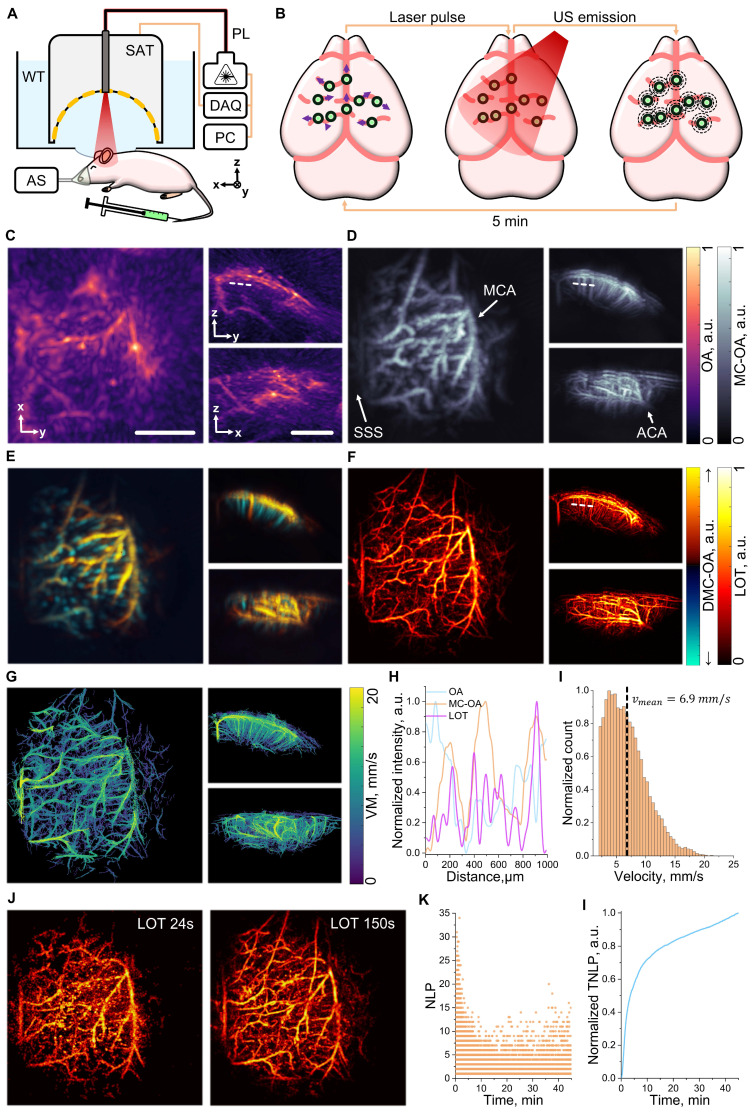
** Optoacoustic tomography (OAT) enhanced with intravenously (i.v.) injected microcapsules (MC)**. (A) Schematic layout of the mouse brain imaging setup. Components include: DAQ (data acquisition system), PL (near-infrared pulsed laser), PC (personal computer), WT (water tank), SAT (512-element spherical array transducer), and AS (anesthesia system). (B) Data acquisition pipeline: MCs injected i.v. circulate throughout cerebral blood vessels and generate OA signals upon excitation with laser pulses, eventually detected by the SAT. (C) Standard OAT image of the murine brain's right hemisphere. Scalebar - 2 mm. (D) Equivalent motion-contrast optoacoustic (MC-OA) image, reconstructed from a compounded sequence of singular value decomposition (SVD)-filtered OAT images. Anatomical structures labeled: SSS (superior sagittal sinus), MCA (middle cerebral artery), and ACA (anterior cerebral artery). (E) Equivalent directional motion-contrast optoacoustic (DMC-OA) image, showing blood flow directions: towards the transducer's spherical surface (↑) and away from it (↓) derived from the OAT sequence. (F) Equivalent localization optoacoustic tomography (LOT) image reconstructed from a point cloud of localized particle positions. (G) Velocity map (VM) of the right hemisphere, reconstructed based on tracking of localized MCs. (H) Line profile comparison from the dashed line in panel (C). (I) Histogram of blood flow velocities derived from the VM data, with a mean velocity of 6.9 mm/s. (J) LOT images reconstructed for acquisition time windows of 24 s and 150 s post-injection. (K) Temporal dependence of the number of localized points (NLP) per frame. (L) Temporal dependence of the normalized total number of localized points (TNLP).

Contrast-enhanced OAT assisted with i.v. injection of individually detectable particles can significantly advance the angiographic imaging performance of standard OAT, offering enhanced resolution and multiparametric visualization of vascular networks both in native 3D 18. The multiparametric imaging performance of MC-based OAT was first tested by imaging the murine brain vasculature. The experimental setup used for this purpose integrates essential components to ensure both optimal imaging performance and physiological monitoring of the animal, with body temperature maintained at 37 °C (Figure 3A, see methods for details). A spherical array transducer (SAT, 512 elements) was used to capture OA signals excited with a NIR nanosecond-duration pulsed laser (PL) coaxially guided through a central aperture of the array. The signals are digitized in real-time with a data acquisition system (DAQ). A water tank, featuring an optically and acoustically transparent membrane at the bottom, was positioned on top of the mouse to ensure acoustic coupling and further facilitate maintaining a uniform mouse body temperature. OAT imaging was performed during and after i.v. injection of a 100 µL bolus of a suspension of MCs (10^8^ particles/mL). The OA signals excited at the MCs enable precise tracking of these within the cerebral vasculature, providing the foundation for detailed vascular imaging (Figure 3B).

OAT images based on endogenous (hemoglobin) contrast were taken as a reference for the capabilities of the standard imaging approach to visualize vascular structures in the right hemisphere of the mouse brain. Key anatomical features, such as the superior sagittal sinus (SSS), the middle cerebral artery (MCA), and the anterior cerebral artery (ACA), were visible in standard OAT images at 800 nm excitation wavelength (Figure 3C). However, accurate imaging of finer vascular networks and penetrating vessels remains challenging. To overcome this limitation, motion-based signal processing via singular value decomposition (SVD)-based filtering was applied. By isolating dynamic signals in a sequence of images generated by moving MCs (Video S1) and compounding the resulting images (see methods for details), motion-contrast optoacoustic (MC-OA) imaging provides a more detailed view of smaller vessels, such as venules and arterioles, across the cortical surface (Figure 3D). These are indistinguishable in conventional imaging arguably due to reduced contrast and limited-view effects 7. The directional motion-contrast optoacoustic (DMC-OA) image further enhances vascular visualization by mapping blood flow directionality (Figure 3E, see methods for details). This approach differentiates between vessels transporting blood toward the SAT, depicted in yellow, and those carrying blood away, shown in blue 38. DCM-OA imaging enables precise mapping of penetrating vessels as well as surface blood flow, offering a more comprehensive and detailed representation of cortical hemodynamics. LOT takes the imaging capabilities a step further by considering a point cloud of localized MC positions to reconstruct super-resolution vascular maps. These maps provide unprecedented detail, revealing microvascular networks previously unresolved by conventional methods, with dimensions in the order of 20 µm (Figure 3F). The signal-to-noise ratio (SNR) is shown to increase by an order of magnitude in MC-OA images relative to conventional OA counterparts. Note that no background is present in LOT images in regions where no particles are detected, resulting in an effective infinite SNR (Figure S3). The improvement in vessel visibility was quantified using AngioTool 0.6a analysis on the maximum intensity projections of the images, demonstrating on average 2.5-fold improvement for such parameters as vessel percentage area (~2.2-fold), total number of junctions (~3.9-fold), total vessel length (~2.4-fold), and total number of endpoints (~1.5-fold) for MC-OA and LOT images with respect to conventional OA counterparts (Figure S4). Particle tracking is then integrated to refine these maps, producing a velocity map (VM) that quantifies blood flow dynamics across the brain (Figure 3G). Further vascular analysis along a selected line profile demonstrates enhanced resolution and improved detection of penetrating vessels (Figure 3H). The VM also highlights a mean blood flow velocity of 6.9 mm/s, showcasing the method's capability to measure cerebral hemodynamics with high precision (Figure 3I).

Temporal analysis of localized MCs revealed a prolonged circulation time, a key feature to ensure precise imaging with a large number of localized particles. Images reconstructed considering acquisitions of 24 and 150 seconds (2400 and 15000 frames) post-injection demonstrate the persistence of signals over time, with a gradual improvement in vascular structure visibility (Figure 3J). Even after 45 minutes of acquisition, up to 10 particles per frame were detected, verifying the extended circulation of MCs in the bloodstream with respect to the droplets used to demonstrate the feasibility of LOT *In vivo* 18. The number of localized points (NLP) per frame underscores this long circulation time (Figure 3K), while the normalized total number of localized points (TNLP) reveals a steady growth over the entire imaging session, further emphasizing the method's potential for long-term vascular monitoring (Figure 3L).

### Mesoscopic imaging of vascularized tissues

**Figure 4:**
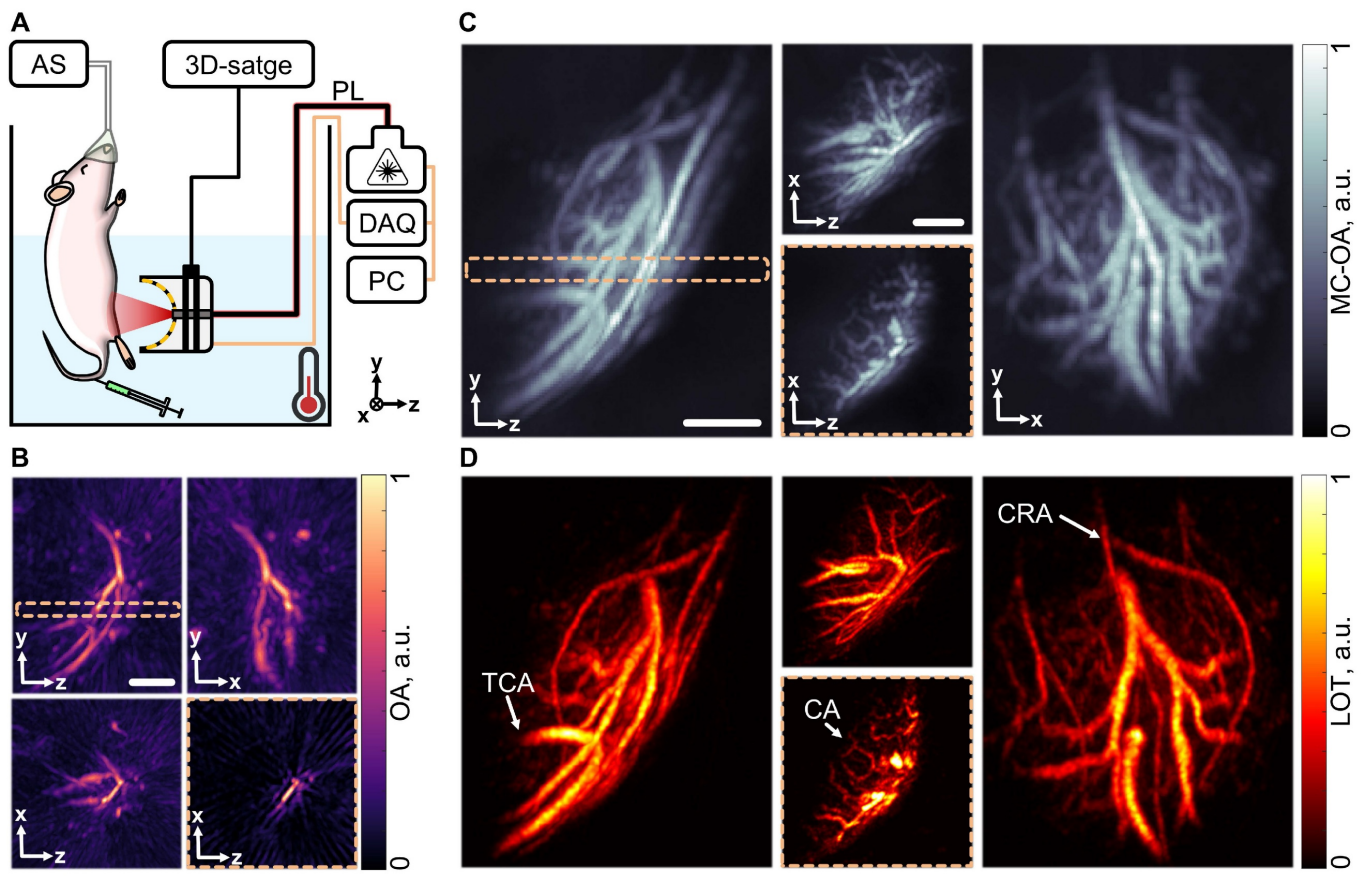
** Mesoscopic imaging of the mouse testis.** (A) Layout of the testicular imaging system: data acquisition system. Components include: DAQ (data acquisition system), PL (near-infrared pulsed laser), PC (personal computer), AS (anesthesia system). (B) Optoacoustic tomography (OAT) image of the right testis of the mouse in different projections. (C) Motion contrast optoacoustic (MC-OA) image of the mouse right testis, reconstructed by compounding a sequence of images of filtered particle motion. (D) Localization optoacoustic tomography (LOT) images of the mouse testis with anatomical structures pointed: TCA (transtesticular centrifugal artery), CA (centripetal arteries), CRA (cremasteric artery). The dashed frame highlights a 400 µm slice through the image. Scalebar - 1 mm.

The performance of MC-enhanced OAT in characterizing subcutaneous vascular networks within the reachable mesoscopic range (millimeter-scale depths) was further validated by visualizing the murine right testis, a representative highly vascularized tissue. For this, the mouse was placed in a heated water tank and a sequence of OAT images of the target region was acquired following i.v. injection of a 100 µL bolus of a suspension of MCs (10^8^ particles/mL, Figure 4A). Standard OAT images revealed prominent vascular structures, such as the transtesticular centrifugal artery (TCA) and a branching vessel in the tunica albuginea. However, internal vascular structures within the testis itself could barely be resolved, as evinced from a slice of the OAT image, taken along the testis' axis (Figure 4B).

To improve visualization, particle motion was filtered, and the data was compiled into an MC-OA image where internal vascular structures became more apparent (Figure 4C). LOT image formation via localization of particles was further performed, which not only highlighted the centripetal arteries supplying the testicular lobules but also revealed the cremasteric artery on the organ's surface (Figure 4D). This significant enhancement in angiographic imaging performance allowed for a clearer view of the testicular vasculature, suggesting that MC-OA imaging and LOT hold great promise as powerful tools for the functional characterization of the reproductive system. These techniques could be used in conjunction with ultrasound imaging to monitor real-time, non-invasive changes in the testicular vasculature, offering a novel approach to reproductive disease research. Currently, real-time, non-invasive imaging techniques capable of visualizing testicular vasculature remain rare, and studies in this area are still limited.

### Visualizing angiogenesis in subcutaneous tumors

**Figure 5:**
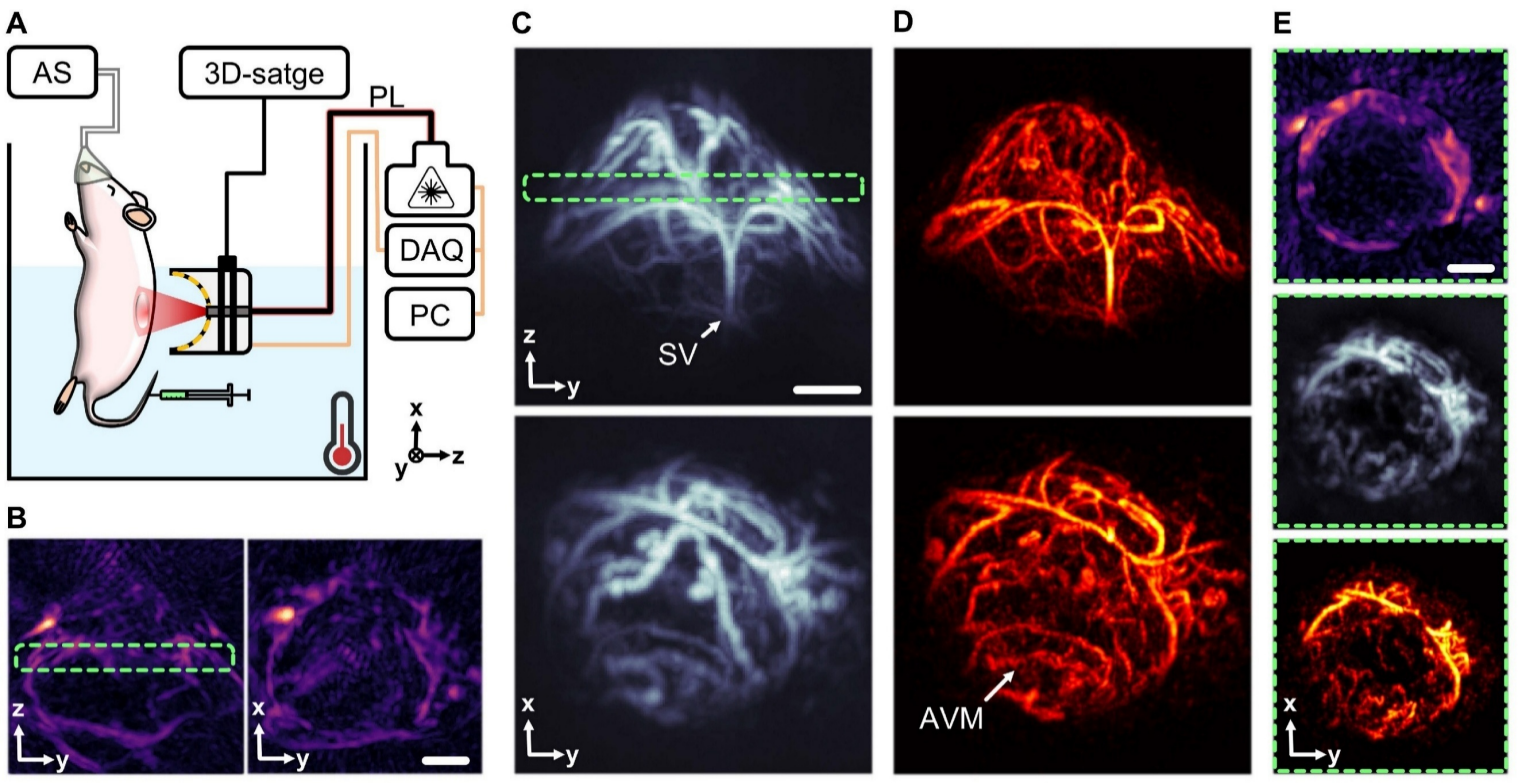
** Mapping neovascularization in a subcutaneous tumor model**. (A) Layout of the subcutaneous tumor imaging setup. Components include: DAQ (data acquisition system), PL (near-infrared pulsed laser), PC (personal computer), AS (anesthesia system). (B) Optoacoustic tomography (OAT) image of a subcutaneous tumor taken 35 days post inoculation of U-87 MG glioma cells. (C) Motion contrast optoacoustic (MC-OA) image of the tumor. (D) Localization optoacoustic tomography (LOT) image of the tumor with anatomical structures pointed: SV (supplying vessel), AVM (arteriovenous malformations). (E) 400 µm thick slices through the tumor image in different modalities, corresponding to a green dashed frame. Scalebar - 1 mm.

Characterization of angiogenesis in subcutaneous tumors is crucial for understanding tumor growth and metastasis, as well as for evaluating the efficacy of novel therapies. The unique angiographic imaging capabilities of MC-enhanced OAT are then poised to play an important role in cancer research. MC-enhanced OAT imaging was performed on a mouse featuring subcutaneous tumors in the right dorsal flank, induced by inoculation of U-87 MG glioma cells. Much like for the testicular imaging experiments, mice were placed in a heated water tank, and sequences of OAT images of the target region were captured following i.v. injection of a 100 µL bolus of an MC suspension (10^8^ particles/mL, Figure 5A). The good penetration depth provided by standard OAT was shown to be effective at highlighting underlying vasculature (Figure 5B), but the reconstructed images lack the resolution needed to visualize the intricate vascular networks within the tumor itself. In contrast, MC-OA (Figure 5C) and LOT (Figure 5D) revealed complex convoluted vascular networks inside the neoplastic lesion, arguably representing neovasculature, formed through angiogenesis. It was possible to identify key structures such as the supplying vessel stem (SV) and, more importantly, highlighting arteriovenous malformations (AVMs) within the tumor mass (Figure 5E). This enhanced visualization can significantly advance our understanding of tumor vascularization and provide a more comprehensive tool for monitoring tumor progression and therapeutic responses.

### Large-scale imaging of the mouse brain

**Figure 6:**
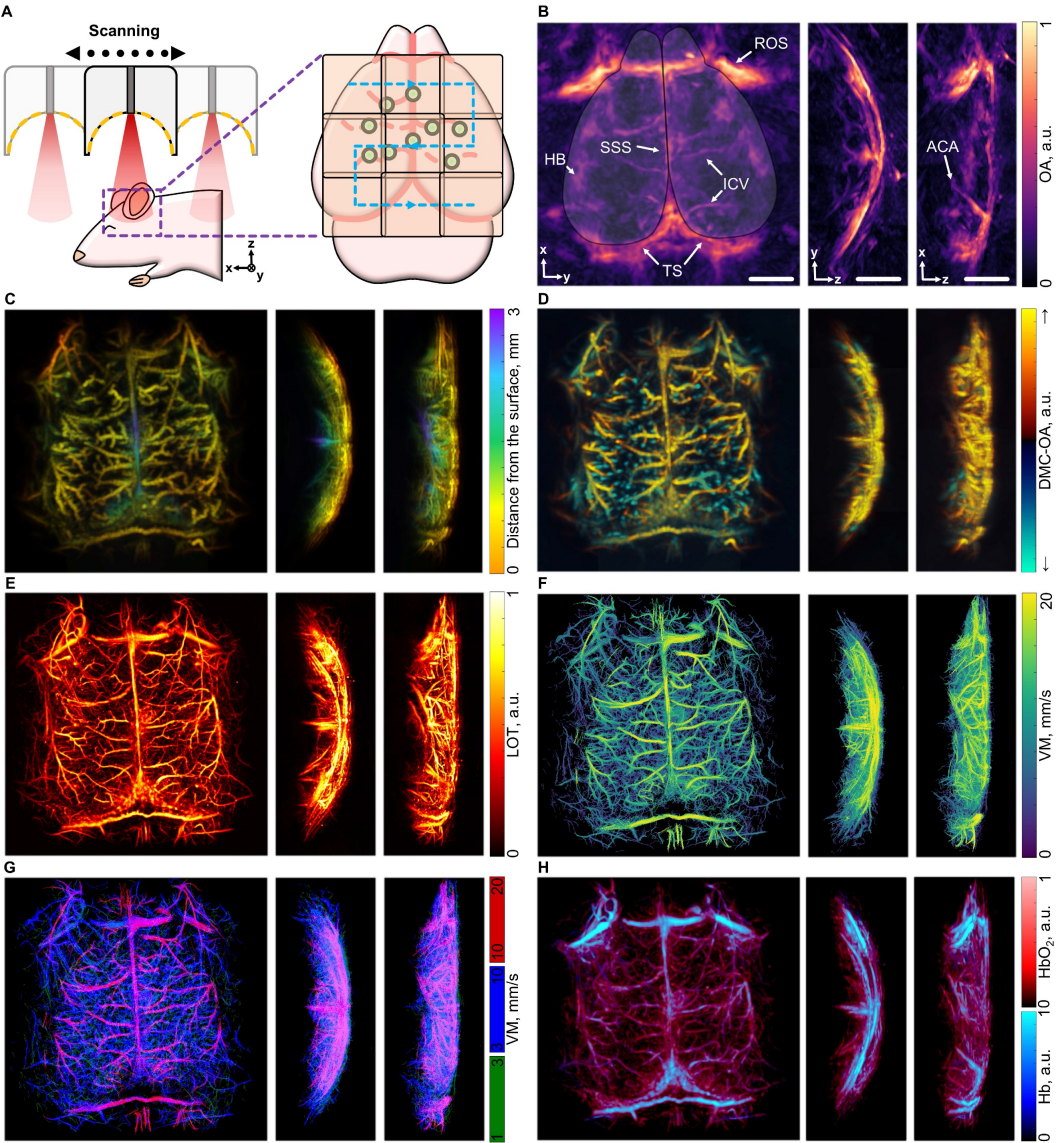
** Panoramic visualization of the mouse brain.** (A) Schematic representation of the multi-position acquisition setup. (B) Optoacoustic tomography (OAT) image of the mouse brain, focusing on depths corresponding to the cortex, acquired from a 9-position raster scan. Anatomical structures labelled: SSS (superior sagittal sinus), ICV (inferior cerebral veins), TS (transverse sinuses), HB (hemispheric border), ROS (retro-orbital sinus) and ACA (anterior cerebral artery). The cortex outline is overlaid on the OA image. (C) Depth-encoded motion contrast optoacoustic (MC-OA) image. (D) Directional motion-contrast optoacoustic (DMC-OA) image, showing the blood flow towards (yellow) and away (blue) from the spherical array transducer. (E) Localization optoacoustic tomography (LOT) image, assembled from the localized positions of the microcapsules (MCs). (F) Velocity map (VM) of the entire cortex. (G) 3-channel split of velocity map showing fast, intermediate and slow velocities in the vessels; (H) Oxygenation map derived from multispectral OAT data masked with LOT data. Scalebar - 2 mm.

The typical field of view (FOV) of the MC-based imaging approaches described in this work is limited to a region where light fluence and the effective sensitivity of the SAT are sufficiently high to enable per-particle detection. Practically, the constrained FOV (typically around 7×7 mm²) of these methods prevents fully encompassing the entire cortex of relatively old mice. Indeed, the cortical size reaches 15 mm in adult mice (older than 10 weeks) 39. To overcome this limitation, we devised a raster scanning approach in which the SAT is systematically repositioned to acquire data from multiple overlapping locations (Figure 6A). This method allows for comprehensive imaging of all cortical structures, spanning from the olfactory bulbs and nasal cavity to the cerebellum. However, the acquisition time is increased when acquiring multiple positions, so the demonstrated long circulation time of the MCs becomes essential to achieve large-scale super-resolution imaging. Whole-brain imaging was achieved by injecting a total volume of 200 µL of a suspension of MCs (10^8^ particles/mL) split into several boluses, representing a viable approach for contrast-enhanced *In vivo* imaging in mice.

Considering the OAT datasets collected across the 9 scanning positions, comprehensive multiparametric imaging could be achieved, providing detailed information that can be displayed in 7 distinct images. First, the standard OAT image highlights primary vascular structures of the brain's cortical layers, including the superior sagittal sinus (SSS), the inferior cerebral veins (ICV), the transverse sinuses (TS), the hemispheric border (HB), and the anterior cerebral artery (ACA) (Figure 6B). The depth-encoded MC-OA image, built by filtering the motion of MCs and applying stacking and masking techniques (see methods for details) further enhances the vascular details, revealing penetrating vessels, the middle cerebral arteries (MCAs), as well as venules and arterioles in the central cortical regions (Figure 6C). The DMC-OA image adds directional information, distinguishing blood flow towards and away from the SAT surface. This offers an effective means of visualizing penetrating vessels extending through the cortex toward the brain ventricles, alongside pairs of vessels carrying blood in the opposite direction toward the cortical surface (Figure 6D). The LOT image further improves resolution and enables detailed visualization of cortical vascular networks (Figure 6E), and subsequent particle tracking generates a full-cortex VM image providing insights into blood flow dynamics (Figure 6F). The map could be separated in 3 channels: 1-3 mm/s, 3-10 mm/s and 10-20 mm/s showing highlighting the vessels in which MC moving fast. The rotating views are available in supplementary materials (Video S2). Additionally, linear unmixing of multispectral (multiwavelength) OAT data enables resolving the biodistributions of oxygenated and deoxygenated forms of hemoglobin within the brain. By combining the intrinsically registered oxygen saturation and LOT images, high-resolution cortical oxygenation maps can be obtained, providing a comprehensive view of the brain's vascular and oxygenation landscape (Figure 6H).

In the current implementation, a complete scan takes up to 45 minutes, but this duration can be significantly decreased by reducing the acquisition time per position. The primary limitation is the laser pulse repetition rate, which governs the speed of data acquisition. Despite these time constraints, the scanning methodology offers immense potential for generating detailed maps of penetrating blood vessels, constructing 3D anatomical references for treatment monitoring, and observing cortex-wide, long-term vascular changes. These vascular changes are critical biomarkers for a range of diseases, including Alzheimer's disease, aneurysm development, ischemia, and stroke.

### Discussion and Conclusions

The MCs developed in this work have been shown to exhibit unique features for enhancing OAT performance. Notably, their strong per-particle signals and prolonged circulation times facilitate the reconstruction of accurate and detailed images of microvascular networks, achieved via compounding of a sequence of OAT frames filtered to isolate signals associated with motion. The OAT data acquired following injection of MCs could be processed to produce multiple image types representing multiparametric contrast. Specifically, MC-OA images were shown to enhance the angiographic imaging performance of standard OAT, with DMC-OA further enabling visualizing blood flow direction. More quantitative insights could be derived from velocity and oxygen saturation maps generated via MC tracking or unmixing of multispectral (multiwavelength) data, respectively. Additionally, the accumulated positions of localized particles could be used to render a LOT image effectively breaking through the acoustic diffraction barrier to reveal functional information on otherwise invisible microvessels.

Biocompatible coreless polyelectrolyte MCs can be further optimized by incorporating biodegradable materials such as poly-arginine, poly-ornithine, chitosan, or combinations with dextran sulfate or alginate 40,41. These biopolymers offer the possibility of tailoring the degradation profiles for specific applications, enhancing biocompatibility, and enabling controlled substance release. Similarly, systems such as BSA/tannic acid can potentially provide additional avenues for tuning the biodegradability and functionality of the MCs 42,43. The overall absorption and OAT signal generation efficiency of the MCs can also potentially be optimized with advanced loading techniques such as freeze-loading 16,44,45. This method allows for an increase in substance loading per capsule while potentially reducing the overall capsule size. Maintaining the absorption per particle at the same level while reducing the size could improve their circulation properties, enhance tissue penetration, and minimize the risks of embolisms potentially produced by particle aggregation. Note, however, that the flexible nature of coreless particle facilitates perfusion through capillaries even for a size comparable to RBCs. Indeed, no capillary arrest was observed when tracking the MCs in a sequence of OAT frames.

MC-based OAT provides a versatile and robust platform for high-resolution vascular imaging and analysis, addressing the limitations of standard OAT. Important performance benchmarks deserve particular attention. For example, limited-view effects are known to be produced when endogenous signals from blood (hemoglobin) are collected with an SAT with less than 180° (2π solid angle) angular coverage, as typically occurs in most practical cases 7. Sparsely distributed microparticles can practically break the continuity of highly concentrated RBCs, resulting in speckled images that can be reconstructed under limited view conditions 12. The MC-OA clearly demonstrates improved angiographic visibility compared to standard OAT under limited view conditions. Indeed, penetrating vertical microvessels in the brain cortex were not visible in the standard OAT because of their orientation relative to the SAT, while these were clearly discerned in the MC-OA image despite being diffraction-limited. The super-resolution angiographic imaging capability of LOT further enabled better resolving these thin vessels, also enhancing limited-view visibility. On the other hand, the demonstrated long circulation time of the MCs enabled large-scale super-resolution imaging, previously hampered by the limited FOV corresponding to sufficient light fluence and sensitivity of the SAT. This paves the wave for a new generation of OAT methods capable of covering multiple spatial and temporal scales with the same type of contrast 46,47. LOT has been demonstrated to reach imaging depths of 3-4 mm with 800 nm excitation wavelength, which are inaccessible with high-resolution optical imaging modalities. Further optimization of materials and a potential shift toward 1064 nm excitation may result in a substantial increase in the depth range being covered. This can make LOT clinically-relevant for visualizing superficial tumors, pathologies in the reproductive system, or microvascular alterations in peripheral vascular diseases, with deeper regions also potentially reachable via endoscopic illumination. Note that the enhanced penetration depth at 1064 nm is not only due to reduced scattering and absorption at this wavelength but also to a higher permissible safety threshold and the availability of laser sources providing high per-pulse energies. Clinical translation can be facilitated with dedicated hardware enabling hybridization of OA and well-established pulse-echo ultrasound imaging 48,49, with the latter also providing super-resolution capabilities at larger depths 50-52.

The proposed methodology combining motion-contrast and localization techniques opens new research avenues for studying cerebral hemodynamics, microvascular morphology, and dynamic vascular changes with unparalleled precision. The imaging performance was demonstrated through examples where high-resolution angiography holds particular importance. Angiographic imaging of the highly vascularized testes provides critical information regarding their health, which is important for diagnosing infertility, varicocele, ischemia, and similar conditions, and adds one more 3D *In vivo* non-invasive imaging modality to existing ultrasound imaging 53-57. Accurate characterization of the brain's vascular structure, function, and potential abnormalities is also essential in neuroscience. MC-based OAT can shed new light onto neurovascular diseases such as stroke, aneurysms, or neurodegenerative conditions by revealing changes in cerebral blood flow and vascular integrity 58,59 Cancer research can also greatly benefit from advanced angiographic imaging tools. These may provide new insights into how tumors develop abnormal blood vessels to support growth and help identify vascular irregularities that impact drug delivery and disease progression 60-63. The theranostic capabilities of the proposed MCs could be further extended to include photothermal tumor therapy and targeted drug delivery, triggered or guided by external fields. For instance, ultrasound, microwave or laser irradiation could be employed to induce the controlled release of encapsulated agents, while an external magnetic field could facilitate the steering or accumulation of MCs within the vasculature of a target region 17,35,64-67. This underscores the high multimodal potential of these LbL-based systems. The inclusion of FeNPs naturally supports MRI as a complementary modality for monitoring the macroscopic distribution of the contrast agent. Additionally, the capsule shell can be engineered to incorporate high-quantum yield, non-aggregating fluorescent dyes or quantum dots for fluorescence imaging or diffuse optical localization imaging, or to use gas-filled microbubbles as the core material to enable ultrasound imaging 68-72.

In conclusion, the proposed MC-based OAT methodology introduces critical new capabilities for enhancing vascular imaging. The long circulation time of MCs is a key factor enabling large-scale super-resolution imaging, which allows for the visualization of microvascular networks across multiple spatial scales with unprecedented detail. This advancement opens new avenues for the detailed analysis of microvascular structures and dynamic changes in blood flow, offering valuable insights into clinically relevant diseases such as neurovascular disorders or cancer.

## Materials and Methods

### Materials

Calcium chloride (CaCl₂), sodium carbonate (Na₂CO₃), poly(diallyldimethylammonium chloride) solution (PDDA, 20%, MW ~200 kDa), poly(sodium styrene sulfonate) (PSS, MW ~70 kDa), indocyanine green (ICG) powder, sodium chloride (NaCl), carboxyl iron oxide nanoparticles (FeNP) with a diameter of 10 nm, and Dulbecco's phosphate-buffered saline (DPBS, without calcium and magnesium chloride) were obtained from Sigma Aldrich. Dulbecco's Modified Eagle's Medium (DMEM), AlamarBlue cell assay kits, fetal bovine serum (FBS), and 96-well plates were purchased from Thermo Fisher Scientific Inc. Chinese Hamster Ovary (CHO) and U87-MG human glioblastoma cells were acquired from CLS Cell Lines Service GmbH. Silicon wafers and standard scanning electron microscopy stubs were obtained from Electron Microscopy Sciences, transparent acoustic coupling gel was sourced from Parker Laboratories Inc. Double-deionized water (DDI, resistivity 18.2 MΩ·cm), produced using a Millipore Milli-Q A10 system, was used in all experiments.

### Microcapsule synthesis

Shell-loaded (coreless) polyelectrolyte microcapsules (MCs) were synthesized using a well-established procedure described previously. First, 1 M aqueous solutions of CaCl₂ and Na₂CO₃, and a 1 mg/mL solution of ICG in DDI were prepared. To form the ICG-loaded CaCO₃ cores, 2 mL of the 1 mg/mL ICG and 2 mL of Na₂CO₃ solution were added to 8 mL of DDI and stirred thoroughly. Next, 2 mL of the CaCl₂ solution was added to this mixture and stirred for 1 minute at 400 rpm using a stirring bar in a dark glass vial. The resulting ICG-loaded CaCO₃ microparticles were then washed four times using centrifugation at 1000 rcf for 1 minute each. Next, 2 mg/mL of a PDDA solution in 0.5 M NaCl was added to the microparticle suspension in an ultrasonic bath. The particles were stirred using a Vortex-Genie 2 shaker (Labgene Scientific SA) for 15 minutes to facilitate the deposition of the PDDA layer. Afterward, the microparticles were rinsed with DDI water three times, and a layer of PSS was applied using the same protocol. This process formed the final multilayered structure: CaCO₃/[PDDA/PSS]₂/[PDDA/FeNP]₄/[PDDA/ICG]₄/[PDDA/PSS]₂. The vaterite cores were subsequently dissolved by the dropwise addition of 1 M HCl (approximately 1 mL) until bubbling stopped. Finally, the MCs underwent five consecutive washing cycles to ensure thorough cleaning.

### Microcapsule characterization

The absorption spectra of the MCs and their components were measured in a 96-well plate (Nunclon, Thermofischer Scientific) using a well-plate reader (M200+, Tecan). Scanning electron microscopy (SEM, Hitachi SU5000) was used to evaluate the morphology and composition of the synthesized particles. Pre-cleaved p-type silicon chips were first cleaned consecutively with acetone, ethanol, and water in an ultrasonic bath. The samples were then drop-casted onto the silicon chips and dried at room temperature. SEM micrographs were obtained at an accelerating voltage of 3 kV.

The biocompatibility of the MCs was assessed using the AlamarBlue assay on Chinese hamster ovary (CHO) cells. The cells were cultured in DMEM supplemented with 10% FBS in a humidified incubator at 37 °C with 5% CO₂. A 96-well plate was seeded with 8×10³ cells per well, each filled with 200 µL of Phenol Red-free cell medium. Suspensions of MCs at varying concentrations (10⁷ to 10¹⁰ particles/mL) were added to separate rows of wells, with one row serving as a control and receiving only PBS. After 24 hours of incubation, the cells were washed with PBS, and 20 µL of AlamarBlue stock solution was added to each well following a standard protocol. The plate was incubated for 4 additional hours, after which 100 µL of the processed medium from each well was transferred to a new 96-well plate to create a reading replica free of cells and residual MCs. Fluorescence was measured using a plate reader (Infinite M200, Tecan) with excitation at 540 nm and emission at 590 nm.

The biosafety of the synthesized MCs was further evaluated in Swiss mice (n = 8 weeks old, 4 males and 4 females). The mice were divided into experimental (n = 4, 2 males and 2 females) and control (n = 4, 2 males and 2 females) groups. The experimental group received a 100 µL intravenous (i.v.) injection of a suspension containing 2×10⁹ particles/mL, while the control group received 100 µL of PBS. Mice were monitored for weight and overall health on days 2, 7, and 11 post-injection before being sacrificed. Hematological analysis was conducted on blood samples collected on day 7 using a BC5000-Vet analyzer (Mindray), and clinical biochemistry was assessed with a VetScan VS2 analyzer (Zoetis) using serum samples collected after sacrifice on day 11. The study complied with Spanish and European regulations and was approved by the Xunta de Galicia.

### Imaging system

A custom-made system was employed for optoacoustic tomography (OAT) imaging. This consisted of a piezoelectric sparse spherical array transducer (SAT) with 512 elements (40 mm curvature radius, 7 MHz central frequency, ~85% full width at half maximum (FWHM) detection bandwidth, 110° angular coverage, Imasonic SaS), previously described in detail 73. The nanosecond-pulsed optical parametric oscillator (OPO)-based Q-switched laser (~7 ns pulse duration, 100 Hz repetition rate, Spitlight EVO-III, Innolas GmbH) delivered light through an 8 mm central aperture in the array via a customized fiber bundle (active output diameter: 5.85 mm, numerical aperture: 0.22, Lightguide GmbH). The transducer array was mounted on a motorized 3-axes XYZ stage (IAI Inc.) within a 3D-printed water tank featuring a central aperture at its bottom covered with polyethylene foil. Ultrasound gel provided acoustic coupling between the imaged medium and the transducer array. Alternatively, the imaged medium was mounted in a water tank, pre-heated to 36 °C and the transducer was translated along 3 axes to locate the imaging position.

Signal reception was handled by a custom data acquisition (DAQ) system capable of parallel digitizing all 512 channels at 40 MSPS rates with 40 dB amplification (Falkenstein Mikrosysteme GmbH). The laser's Pockels cell synchronization signal triggered the DAQ, initiating acquisition with a delay of ~20 µs. The acquired data formed a 3D matrix 
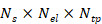
, where 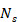
is the number of collected samples (493 samples), 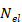
is the number of elements of the spherical array (512 elements), and 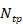
is the total number of frames collected (typically 30000 frames over 5 minutes at 100 Hz).

### Image reconstruction and processing

#### Optoacoustic image reconstruction

Reconstruction of OAT images was performed using a graphics processing unit (GPU)-based back-projection algorithm 74. The reconstruction targeted a volume of interest (VOI) represented as a Cartesian grid with a voxel size of 40 µm. The geometrical parameters of the VOI, such as the reconstruction center and dimensions, were customized for each experiment. The reconstructed OA image at the *i*-th voxel of the grid (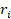) was calculated as 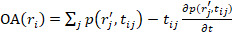, where 
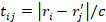
the time of flight between the *i*-th voxel of the grid and the location of the *j*-th element of the spherical array (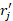). Before reconstruction, the signals were band-pass filtered between 0.2 and 8 MHz.

#### Motion-contrast optoacoustic imaging

The raw signals were initially filtered using a bandpass Butterworth filter with cut-off frequencies of 0.2-8 MHz (Figure S3). Noise arising from slow tissue and breathing motion was then suppressed using a single-value decomposition (SVD) filter. For this, the Casorati matrix, comprising 1200-frame subsets with dimensions (493 samples × 512 transducer elements) x 1200 frames was processed by retaining eigenvectors in the range of 41-1200, which corresponded to signals from fast-moving MCs. Following this, the overall DC offset was removed from each 128-element transducer section, representing 90° angular sectors. The filtered signals were reconstructed into 3D frames with a resolution of 40 µm/pixel and temporally summed over the entire acquisition to produce the motion contrast optoacoustic (MC-OA) image. The depth color-coding was done by assigning the distance in mm according to the reconstructed voxel size relative to the manually segmented surface of the imaged tissue.

#### Directional motion contrast optoacoustic imaging

Acquired OA datasets recorded after injection of MCs were further processed to extract bi-directional blood flow information (towards and away from the spherical array transducer). This was achieved by modifying an algorithm from the literature 38,75,76. The dataset was divided into subsets of dimensions (493 samples × 512 elements × 1200 frames). For each subset, the Hilbert transform was applied to all signal frames (493 samples × 512 elements) along the sample direction to compute a complex analytical signal 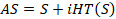, where 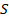
is an initial signal (493 samples long) and 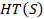
is its Hilbert transform. This removed the Hermitian symmetry of the signal.

Next, a shifted Fast Fourier Transform (FFT) was done and the resulting signal was split into two components 
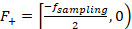
and 
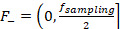
, corresponding to flow towards and away from the transducer elements, respectively (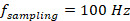, the repetition rate of the laser). These components were shifted and transformed back into the time domain via inverse FFT, producing two signal matrices.

The matrices were subsequently filtered using an SVD filter, reconstructed, and summed to generate the directional motion contrast optoacoustic (DMC-OA) images, representing MC flow towards and away from the array.

#### Localization optoacoustic tomography

Localization optoacoustic tomography (LOT) was performed as the second step in the data processing pipeline to achieve super-resolution imaging of microvascular networks. Following SVD filtering, local intensity maxima, tentatively attributed to signals from MCs, were detected in each 3D frame. A weighted centroid approximation was used for this detection, with an upper limit of 30 localized points per frame. Regions centered on these maxima were then cross-correlated with an empirically measured point spread function (PSF) of the imaging system, retaining only those maxima with correlation coefficients above 0.7. For each validated point, the position was further refined by sub-voxel localization based on the PSF fitting, enhancing spatial accuracy. The LOT image was subsequently generated by overlaying a cloud of localized points corresponding to all detected microcapsules across time frames.

#### Velocity mapping

Particle imaging velocimetry-based blood velocity quantification was conducted as the third step in the processing pipeline to analyze microvascular flow dynamics. Localized points were assessed and linked into tracks using the Munkres algorithm, which optimized the association of points between successive frames. A linking distance of 0.5 mm and a minimum track length of 60 points were used, allowing the detection of blood flow velocities up to a maximum of 50 mm/s, constrained by the imaging frame rate (simpletracker.m available on MathWorks ©Jean-Yves Tinevez, 2019, wrapping Matlab Munkres algorithm implementation of ©Yi Cao 2009). The velocity of each localized point was calculated based on its displacement across frames, and the resulting data was mapped in three dimensions to generate a detailed velocity map (VM) of the cortex.

#### Image stitching

Large-scale mouse brain imaging was done by step-and-go scanning of the SAT along a Cartesian grid with 3 mm step size. Specifically, the collected raw OAT data corresponded to 493 samples × 512 elements × 3 longitudinal positions × 3 transversal positions. The reconstructed 3D volumes were then translated to the corresponding scan positions using the MATLAB function *imtranslate*, and the max compounding was performed to create a complete image.

#### Multi-spectral unmixing

Multispectral unmixing of OAT data was performed as follows. The acquired signals corresponding to different excitation wavelengths were first normalized based on the laser energy transmitted through the fiber bundle and water tank. This was measured using a pyroelectric sensor (Coherent, Inc.) in the range of 700 to 900 nm with 10 nm step sizes. Images for each wavelength were then reconstructed as described earlier and normalized using the estimated wavelength-dependent optical fluence distribution. Fluence was estimated assuming an exponential decay with depth, with the decay constant given by the effective attenuation coefficient 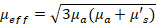, where 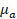
and 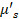
represent the absorption and reduced scattering coefficients, respectively. These coefficients were approximated as uniform throughout the entire volume. The reduced scattering coefficient for brain tissue was taken from the literature. The biodistribution of oxyhemoglobin (HbO₂) and deoxyhemoglobin (Hb) was unmixed using linear spectral fitting of the normalized images to their corresponding spectra. The absorption spectra for both oxy- and deoxyhemoglobins were sourced from the literature. Then the binary mask was created with the LOT data and the oxygenation data was combined with the mask to form the final image.

### *In vivo* imaging experiments

#### Animal models

Animal experiments were conducted following the Swiss Federal Act on Animal Protection and were approved by the Cantonal Veterinary Office Zurich. Four Athymic nude mice (Foxn1nu, Charles River Laboratories, USA, aged 16-26 weeks) were included in the imaging study. The mice were housed in individually ventilated, temperature-controlled cages under a 12-hour dark/light cycle, with pelleted food (3437PXL15, Cargill) and water available *ad libitum*. For tumor induction and imaging, the animals were anesthetized with isoflurane (5% v/v for induction and 1.5% v/v for maintenance, Abbott, Cham, Switzerland) delivered in an oxygen/air mixture (100/400 mL/min).

#### Optoacoustic imaging of the brain

A male athymic nude mouse (16 weeks old) was used for the *In vivo* brain imaging experiment. The mouse was placed on a heating pad to maintain a constant body temperature (37 °C), with its head secured on a stereotactic frame (SGM-4, Narishige). Physiological parameters, including heart rate, respiratory rate, and blood oxygenation, were monitored using a PhysioSuite system (Kent Scientific Corp.). Additional details about the imaging setup are provided in the “Imaging system” section of the “Materials and Methods.” During the scan, a total of 100 μL of a suspension of MCs (10^8^ particles/mL) was injected into the tail vein in 20 µL increments over 5 minutes.

#### Optoacoustic imaging of testis

To image the testis vasculature, a 26-week-old male mouse was positioned in a preheated water tank, and the left testis, specifically the testicular artery, was located using a transducer mounted on motorized stages. During the scan, a total of 100 μL of a suspension of MCs (10^8^ particles/mL) was injected into the tail vein in 20 µL increments over 5 minutes. The motion of the MCs was observed shortly after injection using a 10 Hz nanosecond OPO laser excitation.

#### Optoacoustic imaging of subcutaneous tumors

The U87-MG human glioblastoma cell line was cultured following the manufacturer's protocol in EMEM medium supplemented with 10% fetal bovine serum (FBS) under a 5% CO₂ atmosphere in a humidified incubator. The cells were detached using trypsin, washed three times with PBS, and re-suspended in PBS. Cell concentration was determined using a hemocytometer (Neubauer chamber, Blaubrand), and adjusted to approximately 10⁷ cells/mL. Mice were anesthetized as described in the “Animal models” section, and 150 µL of the cell suspension, containing 2 × 10⁶ cells, was injected subcutaneously into the lumbar region, which had been pre-cleaned with a surgical wipe soaked in 70% ethanol. The mice were monitored over five weeks, during which the tumors grew to approximately 75 mm³. Imaging was conducted following the same protocol outlined in the “Optoacoustic imaging of testis” section.

#### Large-scale brain imaging

A 16-week-old male athymic nude mouse was used for large-scale *In vivo* OAT brain imaging. The mouse was placed on a heating pad to maintain a constant body temperature (37 °C), with its head secured on a stereotactic frame (SGM-4, Narishige). Physiological parameters, including heart rate, respiratory rate, and blood oxygenation, were monitored using a PhysioSuite system (Kent Scientific Corp.). Multispectral (multiwavelength) OAT imaging was conducted across 21 optical wavelengths (700-900 nm in 10 nm steps), with each wavelength averaged 50 times. To obtain comprehensive brain coverage, a 9-position raster scan was performed with uniform 3 mm steps along the X and Y axes. A 200 μL suspension of MCs (10^8^ particles/mL) was injected into the tail vein in 20 µL increments at each acquisition position under a single excitation wavelength of 800 nm. The signals from the moving MCs were collected over 5 minutes per position (30000 frames per position), ensuring full brain imaging coverage.

## Supplementary Material

Supplementary figures and tables.

Supplementary video 1.

Supplementary video 2.
