# Supplementary material for: Large-scale super-resolution optoacoustic imaging facilitated by FeNP/ICG-loaded coreless polyelectrolyte microcapsules
Source: Theranostics. 2025 May 25;15(13):6412–27. doi: 10.7150/thno.112050 (PMC12160020; doi:10.7150/thno.112050)
Supplement: Supplementary file 1 — Supplementary figures and tables. [file thnov15p6412s1.pdf]

Supplementary Information

Large-scale super-resolution optoacoustic imaging facilitated by FeNP/ICG-loaded coreless polyelectrolyte microcapsules

Daniil Nozdriukhin [1,2], Shuxin Lyu [1,2,3], Daniel Razansky [1,2,*], Xosé Luís Deán-Ben [1,2,*]

[1]Institute for Biomedical Engineering and Institute of Pharmacology and Toxicology, Faculty of Medicine, University of Zurich, Winterthurerstrasse 190, Zurich, 8057 Switzerland

[2]Institute for Biomedical Engineering, Department of Information Technology and Electrical Engineering, ETH Zurich, Wolfgang-Pauli-Strasse 27, Zurich, 8093 Switzerland

[3]Institute of Medical Technology, Shanxi Medical University, 030001 Taiyuan, China

[*]Corresponding authors: Daniel Razansky, E-mail: daniel.razansky@pharma.uzh.ch. Xosé Luís Deán-Ben, E-mail: xl.deanben@pharma.uzh.ch

Supplementary figures

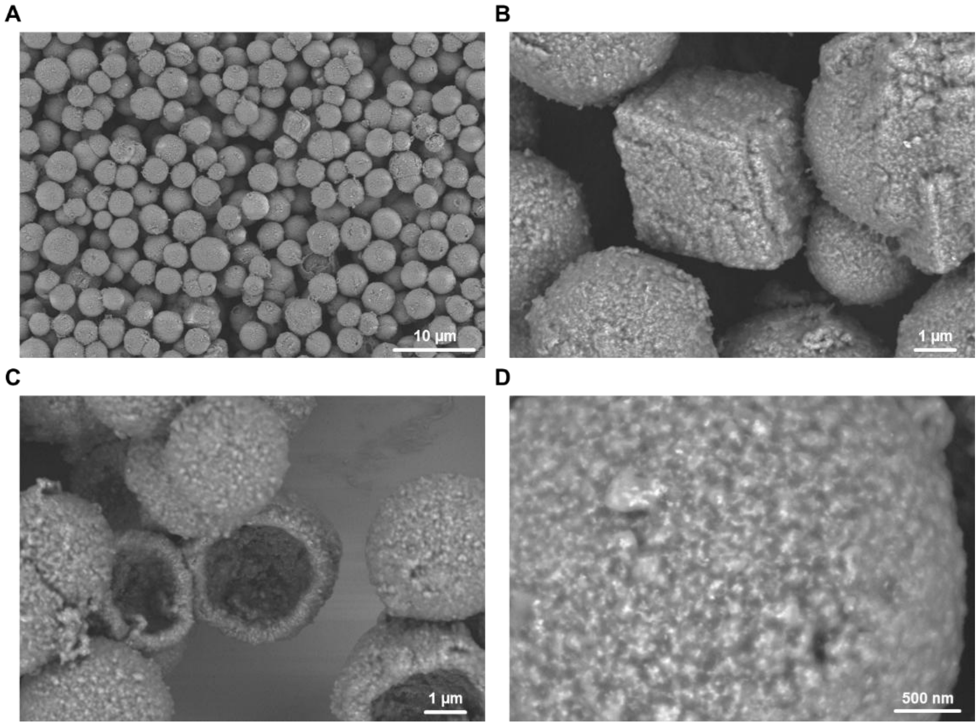

Figure S1. (A) Scanning electron microscopy (SEM) image of the coated $CaCO_3$ microparticles before the core dissolution. (B) Example of recrystallized microparticle with disrupted shell. (C) Close view of the microcapsules (MCs), the top wall caved in because of drying during the sample preparation procedure. (D) Zoomed-in view of the MC's surface. All images were acquired in BSE mode.

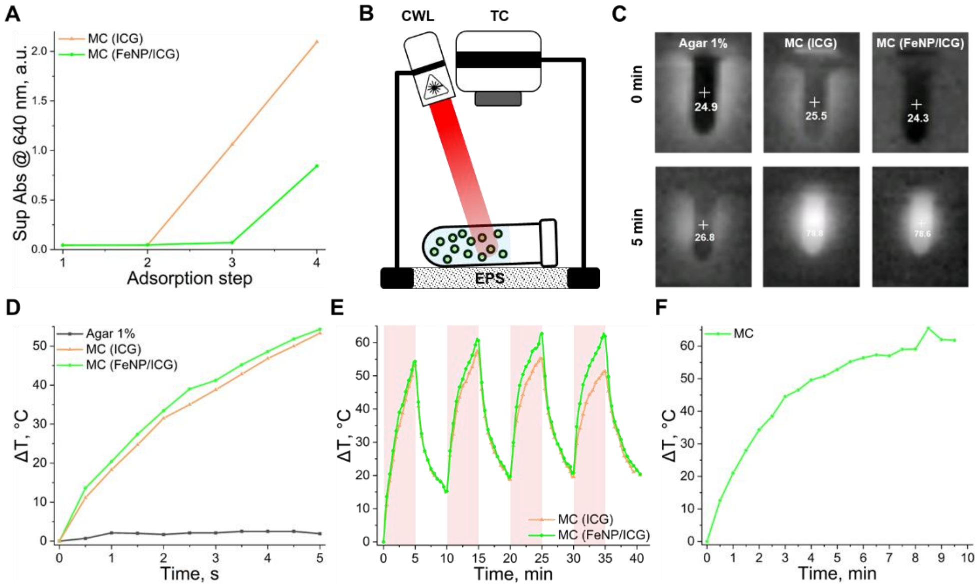

Supplementary Figure S2. (A) Supernatant absorbance after ICG adsorption steps in FeNP-containing and blank microcapsules (MCs). (B) Scheme of the photothermal experiment: CWL – collimated continuous wave laser operating at 795 nm with 1 W/cm^2 light fluence, TC – thermal camera, EPS – expanded polystyrene screen. (C) Thermal images of pure 1% Agar gel, 1% Agar gel with 10^8 mL^{-1} MCs containing ICG, and 1% Agar gel with 10^8 mL^{-1} MCs containing FeNP and ICG. All samples were embedded in 2 mL tubes and exposed to laser irradiation for 5 minutes. (D) Photothermal heating curve of the aforementioned substances. (E) Photothermal cycling of the ICG-containing and FeNP/ICG-containing MCs. Red regions indicate the heating phase and white regions indicate cooling phase at room temperature with the laser illumination being switched off. The signal from ICG-containing MCs degrades at the third cycle. (F) Photothermal heating curve, demonstrating the maximal temperature rise reached in the presented conditions with FeNP/ICG-containing microcapsules, distributed in 1% Agar gel.

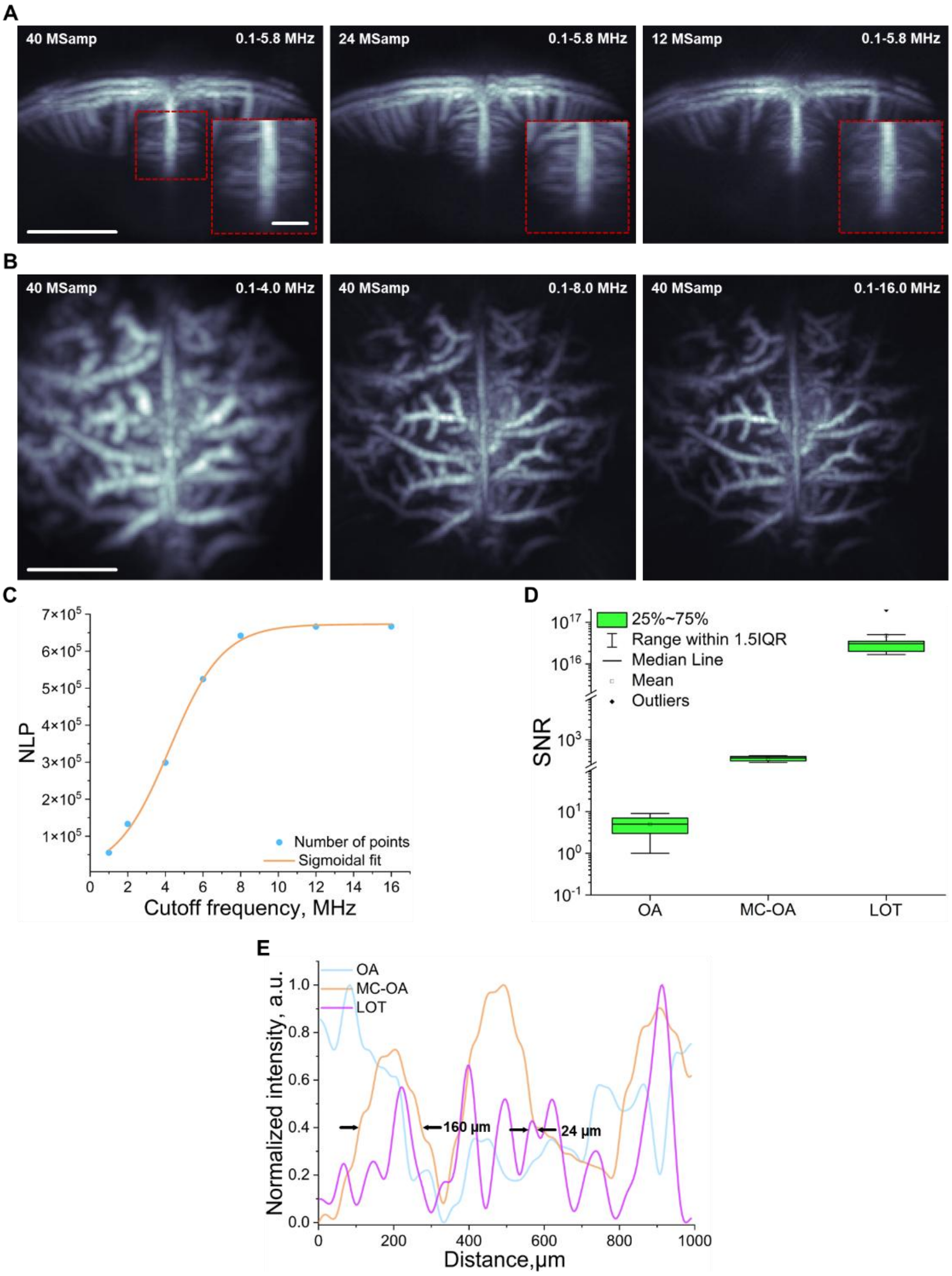

Figure S3. (A) Reconstructed motion-contrast optoacoustic (MC-OA) images, acquired with 40, 24, and 12 MSamp/s sampling rates, showing the degradation of the vascular image at 12 MSamp/s and almost no change at 24 MSamp/s. (B) Reconstructed MC-OA images with high cut-off frequencies of 4, 8 and 16 MHz, showing the resolution increase with an increase of a cut-off frequency for MC-OA. (C) Dependency of the number of localized points (NLP) on the cut-off frequency, showing almost no difference after 8 MHz, that was used as a high-frequency filter in this study. Scalebar – 2 mm. (D) Calculated SNR values for 9 datasets of the mouse brain imaging for conventional optoacoustic (OA), MC-OA and localization optoacoustic tomography (LOT). The signal was taken in the vessel region, the noise was taken outside of the brain area. (E) The profiles along the penetrating vessels with marked-up detectable feature sizes for MC-OA and LOT. The SNR for LOT is probably due to eps in Matlab (minimum real number) since the background is 0, or eps if the images are single or double.

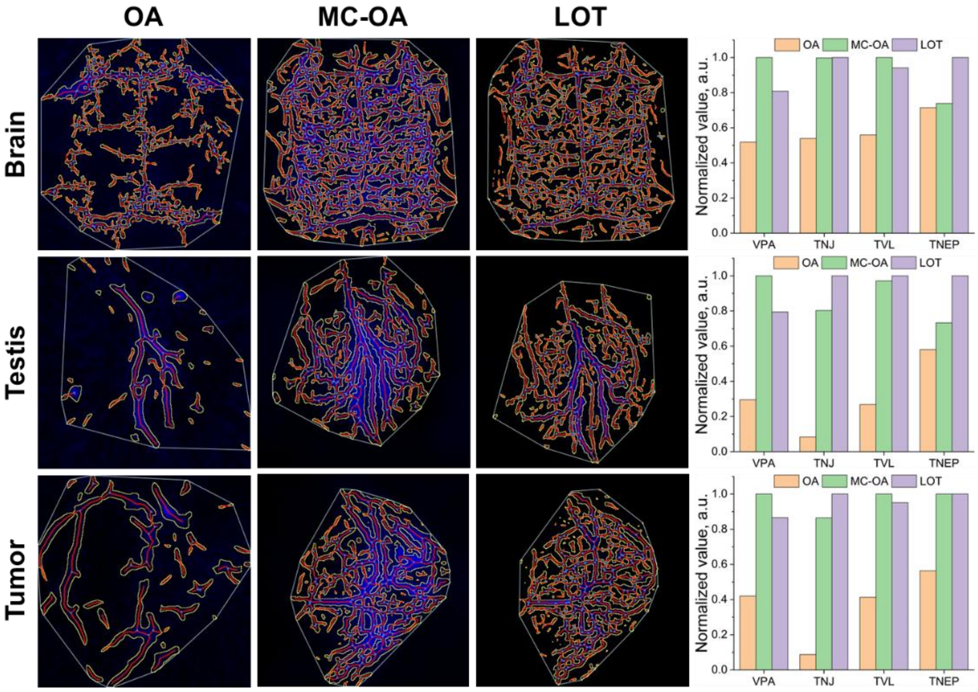

Supplementary Figure S4. Vessel quantification of conventional optoacoustic (OA), motion-contrast optoacoustic (MC-OA) and localization optoacoustic tomography (LOT) images performed with AngioTool 0.6a: vessel percentage area (VPA), total number of junctions (TNJ), total vessel length (TVL), and total number of endpoints (TNEP) are indicated.

Supplementary videos

Video S1. 3-projection optoacoustic tomography (OAT) image of the moving microcapsules after the injection.

Video S2. Rotating 3D-views of the mouse brain vasculature acquired with optoacoustic tomography (OAT), motion-contrast optoacoustic imaging (MC-OA), directional motion contrast optoacoustic imaging (DMC-OA), localization optoacoustic tomography (LOT), along with blood flow velocity mapping (VM) and velocity splitting (VMS).
